# Opposite Regulatory Effects of Immobilized Cations on the Folding Vs. Assembly of Melittin

**DOI:** 10.3389/fchem.2021.685947

**Published:** 2021-06-11

**Authors:** Lanlan Yu, Zhun Deng, Wenbo Zhang, Shuli Liu, Feiyi Zhang, Jianjian Zhou, Chunhua Ma, Chenxuan Wang

**Affiliations:** ^1^ State Key Laboratory of Medical Molecular Biology, Chinese Academy of Medical Sciences and Peking Union Medical College, Institute of Basic Medical Sciences, Beijing, China; ^2^ Department of Clinical Laboratory, Peking University Civil Aviation School of Clinical Medicine, Beijing, China; ^3^ Institute for Advanced Materials, Jiangsu University, Zhenjiang, China; ^4^ Unchained Labs, Shanghai, China

**Keywords:** self-assembly, protein folding, peptide, hydrophobic interactions, charge-related interactions

## Abstract

Ions are crucial in modulating the protein structure. For the free ions in bulk solution, ammonium is kosmotropic (structure forming) and guanidinium is chaotropic (structure breaking) to the protein structure within the Hofmeister series. However, the effect of immobilized ions on a protein surface is less explored. Herein, we explored the influence of two immobilized cations (ammonium in the side chain of lysine and guanidinium in the side chain of arginine) on the folding and assembly of melittin. Melittin adopts an α-helix structure and is driven by hydrophobic interactions to associate into a helical bundle. To test the influence of immobilized cations on the peptide structure, we designed the homozygous mutants exclusively containing ammonium (melittin-K) or guanidinium (melittin-R) and compared the differences of melittin-K vs. melittin-R in their folding, assembly, and molecular functions. The side chains of lysine and arginine differ in their influences on the folding and assembly of melittin. Specifically, the side chain of R increases the α-helical propensity of melittin relative to that of K, following an inverse Hofmeister series. In contrast, the side chain of K favors the assembly of melittin relative to the side chain of R in line with a direct Hofmeister series. The opposite regulatory effects of immobilized cations on the folding and assembly of melittin highlight the complexity of the noncovalent interactions that govern protein intermolecular architecture.

## Introduction

The folding and assembly of proteins by intra- and intermolecular interactions are fundamental for building a variety of protein machines and structural scaffolds and uncovering a molecular mechanism for protein misfolding diseases (Patel et al., 2012; Pace et al., 2014; Meuzelaar et al., 2016; Du et al., 2019; Zhang et al., 2020; Yu et al., 2021). Protein folding and assembling behaviors are not conceptually dependent on the energetic summation over pairwise amino acid contacts, which can be semiempirically predicted from the primary structure, but also the interactions with solvents and salts in the surrounding environment (Acharya et al., 2010; Garde, 2015; Ma et al., 2015; Wang et al., 2017). Specifically, the ubiquitous salts in solution have a profound influence on affecting protein–protein interactions and the subsequent association states, which is known as the Hofmeister effect (Kunz et al., 2004; Vlachy et al., 2009; Zhang and Cremer, 2010). Substantial efforts have been made to investigate the effects of free ions on protein assembly, whereas cations or anions are ranked as an empirical Hofmeister series and classified into kosmotropes (protein structure forming and salting out) and chaotropes (protein structure breaking and salting in) (Kunz et al., 2004; Vlachy et al., 2009; Zhang and Cremer, 2009, 2010; Metrick et al., 2019). However, the effect of immobilized ions on a protein surface, although of equal importance, is less explored (Huang et al., 2015; Ma et al., 2015; Wang et al., 2021). The immobilized kosmotropic ion (e.g., ammonium in the side chain of lysine, K) and chaotropic ion (e.g., guanidinium in the side chain of arginine, R) are widely distributed in the proximity of a nonpolar patch across native protein structures, such as membrane-active peptides and coiled-coil architectures(Santo and Berkowitz, 2012; Thomas et al., 2013; Pino-Angeles and Lazaridis, 2018; Zhang et al., 2020). Elucidation of the impact of immobilized ions on protein structures and stability is directly contributed to the design and manipulation of protein assembly and hydration behaviors (Biok et al., 2019; Wang et al., 2021).

**FIGURE 1:**
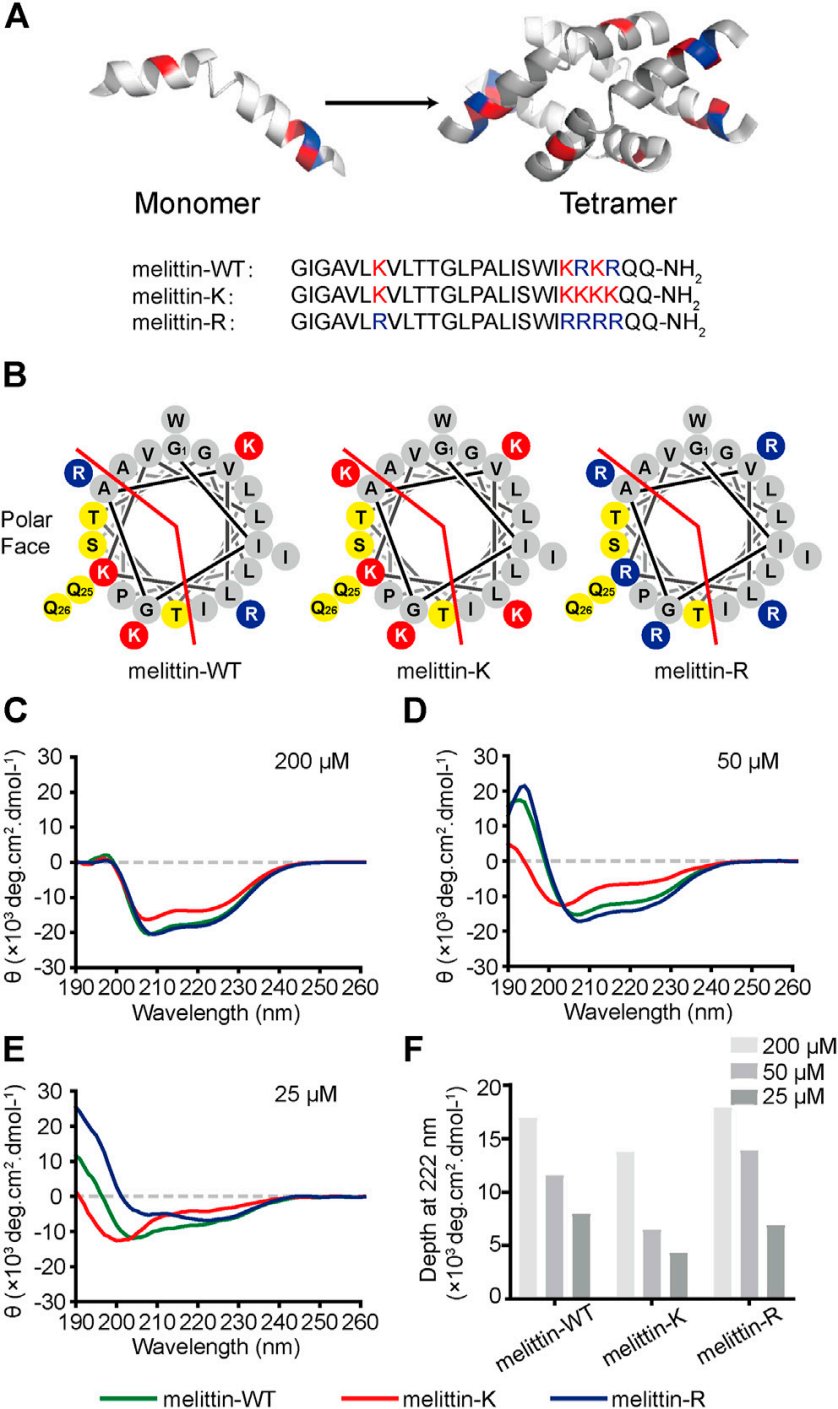
Impacts of K vs. R on the folding structure of melittin. **(A)** Lateral view of a globally amphiphilic melittin-WT peptide and the peptide sequences of melittin-WT, -K, and -R. **(B)** The helical wheel diagrams of melittin peptides, showing the nonpolar and polar faces of the helix, separated by the red line. The solid red discs with a K letter sign depict the side chains of lysine amino acid residues, while the solid blue discs with an R letter represent the ones of arginine residues. The yellow discs show the polar-uncharged residues and gray discs represent the nonpolar ones. **(C–E)** The CD spectra of melittin peptides at 200 μM **(C)**, 50 μM **(D)**, and 25 μM **(E)**. **(F)** The depth of molar ellipticity of melittin peptides at 222 nm.

We previously evaluated the impact of immobilized ions on the hydrophobic interactions and assembly of a globally amphiphilic β-amino acid peptide (β-peptide) (Ma et al., 2015; Wang et al., 2021). In this study, we selected melittin, a naturally occurring peptide derived from honeybee venom, as a model native system to evaluate the influences of immobilized cations on protein folding and assembly structures. Wild-type melittin (melittin-WT) is a cationic 26-residue peptide with six positive charges (i.e., the side chains of K and R and the N terminus) at physiological conditions (Figure 1A; Bechinger, 1997). Melittin maintains a random coil monomeric state in a dilute aqueous solution and switches into an α-helical tetrameric state in the solution of high peptide concentration (Raghuraman and Chattopadhyay, 2006; Othon et al., 2009). The α-helical conformation renders melittin with an amphiphilic surface chemical pattern (Figures 1A,B) to exhibit strong membrane lytic activity against Gram-positive and Gram-negative bacteria, erythrocytes, and cancer cells (Raghuraman and Chattopadhyay, 2006; Othon et al., 2009; Kurgan et al., 2019). Taken together, the melittin system and the previous β-peptide system share an amphiphilic feature that a nonpolar domain is adjacent to cationic side chains. But these two systems differ in the conformational stability, that is, β-peptide possesses a fixed helical conformation (Ma et al., 2015; Wang et al., 2021), whereas the helical propensity of melittin is dependent on peptide concentration.

Wild-type melittin is a heterozygote containing both kosmotropic lysine and chaotropic arginine. We designed and synthesized two sequence mutants, melittin-K and melittin-R, which exclusively include either lysine or arginine residues (Figures 1A,B). Such an approach promises us to explore the influences of immobilized kosmotropic vs. chaotropic cations, that is, ammonium vs. guanidinium, on the folding and association behaviors of melittin, respectively. We employed circular dichroism (CD) to investigate the folding structure of melittin-WT, -K, and -R. Photoinduced cross-linking of unmodified proteins (PICUP) method was combined with sodium dodecyl sulfate–polyacrylamide gel electrophoresis (SDS-PAGE) to determine the assembly states of each peptide mutants in solution at a neutral solution(Bitan and Teplow, 2004). To provide additional insights into the impacts of immobilized ammonium vs. guanidinium on the molecular recognition of melittin, we further compared the interactions of melittin-WT/-K/-R with a variety of phospholipids, bacteria, and mammalian cells.

## Materials and Methods

### Materials

Synthetic melittin-WT/-K/-R peptides (lyophilized powders) were purchased from Bankpeptide Biological Technology Co., Ltd. The purity of peptide powders is above 98%, which was verified by high-performance liquid chromatography and mass spectroscopy. Related reagents (analytical grade) in the experiments were purchased from commercial vendors as follows: 1,1,1,3,3,3-hexafluoro-2-propanol (HFIP, Innochem), 1,6-Diphenyl-1,3,5-hexatriene (DPH, Aladdin), Tris (2,2′-bipyridyl) dichlororuthenium (II) hexahydrate (RuBpy, Macklin), ammonium persulfate (APS, from BBI), dithiothreitol (DTT, from BBI), and Tris, Tricine (from BBI). 1-Palmitoyl-2-hydroxy-*sn*-glycero-3-phosphate sodium salt (LysoPA), 1-palmitoyl-2-oleoyl-*sn*-glycero-3-(phospho-*rac*-(1-glycerol)) sodium salt (POPG), 1,2-dipalmitoyl-*sn*-glycero-3-phosphocholine (DDPC), and D-*erythro*-dihydrosphingosine (sphinganine) were purchased from Avanti. Assay kits and commercial vendors are listed as follows: MTT cell proliferation colorimetric assay kit (Cat.#M1020, Solarbio), Tricine–SDS-PAGE gel preparation kit (Cat.#C641100, BBI Life Science Corporation), penicillin/streptomycin (Invitrogen), Fetal bovine serum (FBS), and DMEM and F12 basic media (Gibco).

### Concentration Measurement

The concentration of melittin peptides in the solution was determined by a UV–Vis spectrophotometer (PerkinElmer, United States). All buffers for peptide solutions were filtered through 0.22-μm nitrocellulose membranes before usage (Millipore). Melittin peptides were dissolved in 6 M guanidine-HCl solution to unfold peptides and measured the UV absorbance at the wavelength of 280 nm. The concentration of peptide solutions was calculated by Beer–Lambert law with the extinction coefficients of 5690 M^−1^ cm^−1^ for the tryptophan in the peptide sequence.

### CD Measurement

The CD spectra were recorded with a circular dichroism spectropolarimeter system (Jasco J-1500, Japan) at room temperature of ∼25° C, using a cuvette with a 0.1-cm path length. Melittin peptides were dissolved in the sodium phosphate buffer (pH = 7.3 and the ionic strength is 0.17 M) with the final concentration of 10, 25, 50, and 200 μM. CD spectra were recorded using a scan speed of 100 nm/min, a digital integration time of 1 s, and a bandwidth of 2 nm. At least two scans with step increments of 1 nm in wavelength were accumulated from 260 to 190 nm for far-UV scans. The phosphate buffer signal was subtracted from the sample spectra. Data were converted to ellipticity (*θ,* deg cm^2^ dmol^−1^) according to the following equation:

[θ] = 1000*Ψ / (nlc),

where Ψ is the CD signal in mdeg, *n* is the number of amino acid residues, *l* is the path length in mm, and *c* is the concentration in mM.

### Photoinduced Cross-Linking of Unmodified Proteins (PICUP)

180 μL of 200 μM peptide 1 × PBS solution was mixed with 10 μL 1 mM RuBpy and 10 μL 20 mM APS. Chemical cross-linking was initiated by the illumination of a xenon UV lamp at 20 A for 1 s and then quenched by adding 10 μL 1 M DTT into the solution. The association state of melittins was subsequently analyzed by sodium dodecyl sulfate–polyacrylamide gel electrophoresis (SDS-PAGE). The proportion fraction of band intensity (*p*_*i*_) in SDS-PAGE pictures was processed and calculated by ImageJ (version 1.53c). Coomassie blue dye was used to stain melittins in SDS-PAGE gels. A mixed solution containing melittin, RuBpy, and APS without illumination was used as a control group.

### Evaluation of Thermal Stability

The stability of melittin peptides against thermal denaturation was investigated by an all-in-one UNcle stability platform (Unchained Labs, Norton, MA). 5 μL of 1 mM melittin samples in 1 × PBS were loaded into each well for the intrinsic fluorescence and static light scattering (SLS) measurements with duplicates under a temperature range from 25 to 95° C.

### Measurement of *CAC*

The *CAC* values were determined using previously reported methods (Chattopadhyay and London, 1984). A fluorescent probe DPH stock solution (2.4 mM DPH in tetrahydrofuran) was diluted to be 6 μM in 1 × PBS solution before use. Two-fold serial dilutions of lipid with or without 12 μM melittin solution were added into a black 96-well plate (Corning, United States) for a total volume of 50 μL in each well. 50-μL aliquots of the 6 μM DPH solution were added to reach 100 μL in each well. These black plates covered with lid were incubated in the dark for 1 h, and the fluorescence intensity (excitation at 358 nm and emission at 428 nm) was recorded using the Synergy H4 microplate reader (BioTek, United States). Every sample had three duplicate wells and the measurement was repeated at least twice to test the reproducibility. The fluorescence intensity of each sample was plotted logarithmically against lipid concentration. Two regression lines were used to describe the low-concentration region with static and low fluorescence and the high-concentration region with distinctly increasing fluorescence, respectively. The *CAC* value was determined from the intersection of these two lines. *CAC* values are reported as the average of duplicate assays.

### Antibacterial Assays

The antibacterial activity for melittin peptides was determined by a broth microdilution method. The bacterial strains used in the antibacterial assays were multiple drug–resistant (MDR) *Acinetobacter baumannii* (*A. baumannii*) 1814516 (clinically isolated from Civil Aviation General Hospital, Beijing) and *A. baumannii* BAA747, and *Staphylococcus aureus* 29231, methicillin-resistant *Staphylococcus aureus* (MRSA) USA-300, MRSA USA-400 (obtained from American Type Culture Collection Bank, ATCC). Bacterial cells were incubated in Luria–Bertani (LB) agar overnight at 37 C, and then a bacterial suspension of approximately 2 × 10^6^ CFU/ml in LB growth medium was prepared. Aliquots (50 μL) were added into each well in sterile 96-well plates (Corning, United States) with 50 μL of medium containing the peptide solutions in two-fold serial dilutions. 50 μL of LB medium was used as a control for all assays. Each well had a total volume of 100 μL and 1 × 10^5^ CFU/well. The plate was incubated at 37° C for 24 h. The optical density (OD) at a wavelength of 600 nm, which determines the bacterial growth, was recorded using the Synergy H4 microplate reader (BioTek, United States). The lowest concentration at which 80% inhibition of bacterial growth is observed is defined as the minimum inhibitory concentration (*MIC*) of peptides. MIC measurements were performed repeatedly for reproducibility.

### Cytotoxicity Assay

The cell lines used in this cytotoxicity assay were purchased from commercial vendors as follows: AGS cells (a human gastric adenocarcinoma cell line, purchased from the Peking Union Medical College Cell Culture Center) and HNEpCs (human nasal epithelial cells, obtained from ATCC). The MTS experiment was performed to test the cytotoxicity of melittin peptides. AGS or HNEpCs were pre-seeded into a transparent 96-well plate at the density of 6,000 cells in each well overnight, and then incubated with two-fold serial dilutions of peptides for 24 h. The culture medium was replaced with 100 µL of MTS solution for each well and incubated for an additional 4 h. Subsequently, we removed the supernatant and added 110 μL DMSO into each well, and vortexed for 10 min. The OD at a wavelength of 490 nm was recorded by using the Synergy H4 microplate reader (BioTek, United States). The viability of the cell incubated with a fresh culture medium defines 100%.

## Results and Discussion

### Influence of K Vs. R on Melittin Folding Structure

Melittin-WT is a heterozygote containing both kosmotropic lysine and chaotropic arginine. Hence, the sequence mutants melittin-K and melittin-R exclusively including either lysine or arginine residues were designed and synthesized to explore the effects of immobilized kosmotropic vs. chaotropic cations, that is, ammonium vs. guanidinium, on the folding structures of melittin, accordingly. We applied a CD spectrometer to explore the folding structures of melittin peptides at different concentrations. At 200 μM, all melittin peptides adopt α-helical structures with two distinctive negative peaks at 208 and 222 nm (Figure 1C). The CD spectra of melittin-WT and -R are almost the same with an ellipticity (*θ*) of ca. −17,500 deg cm^2^ dmol^−1^ at 222 nm. In contrast, melittin-K exhibits weaker α-helical propensity with a minimum of −13,700 deg cm^2^ dmol^−1^ at 222 nm. To investigate the dependence of helical propensity on melittin concentration, we measured the CD spectra of melittin peptides at 50, 25, and 10 μM (Figures 1C–F; Supplementary Figure S1). As the concentrations of melittin peptides decreased, the molar ellipticity at 222 nm (the characteristic feature of α-helix) was reduced, suggesting the dilution unfolds the α-helical conformation of melittin (Figure 1F). The identity of positive charge changes the potential of melittin to maintain its helical structure against the dilution of peptide concentration. Melittin-K switched from α-helix to a random coil when the concentration decreased from 50 to 25 μM. In contrast, melittin-WT and -R maintained the α-helical structure at 25 μM (Figures 1D,E). At 10 μM, all melittin peptides adopted random coil (Supplementary Figure S1). According to a previous study, the [*θ*]_222_/[*θ*]_208_ ratio is an indicator for the helical propensity of a peptide, in which >1.0 is indicative of an ordered, helical protein chain, and <1.0 suggests disordered helices (Sanavio et al., 2007). The [*θ*]_*222*_ */*[*θ*]_*208*_ value of melittin-R (1.29) is significantly higher than that of melittin-WT (0.74) at the concentration of 25 μM (Table S1). One would predict that melittin-R might have a lower critical concentration relative to melittin-WT. Our CD experiments lead to an observation that the side chain of arginine facilitates the helical folding of melittin relative to the side chain of lysine. The rank of lysine vs. arginine influence on peptide folding is opposite to the Hofmeister series for free cations, in which ammonium is structure forming rather than a guanidinium.

### Influence of K Vs. R on the Association of Melittin

**FIGURE 2:**
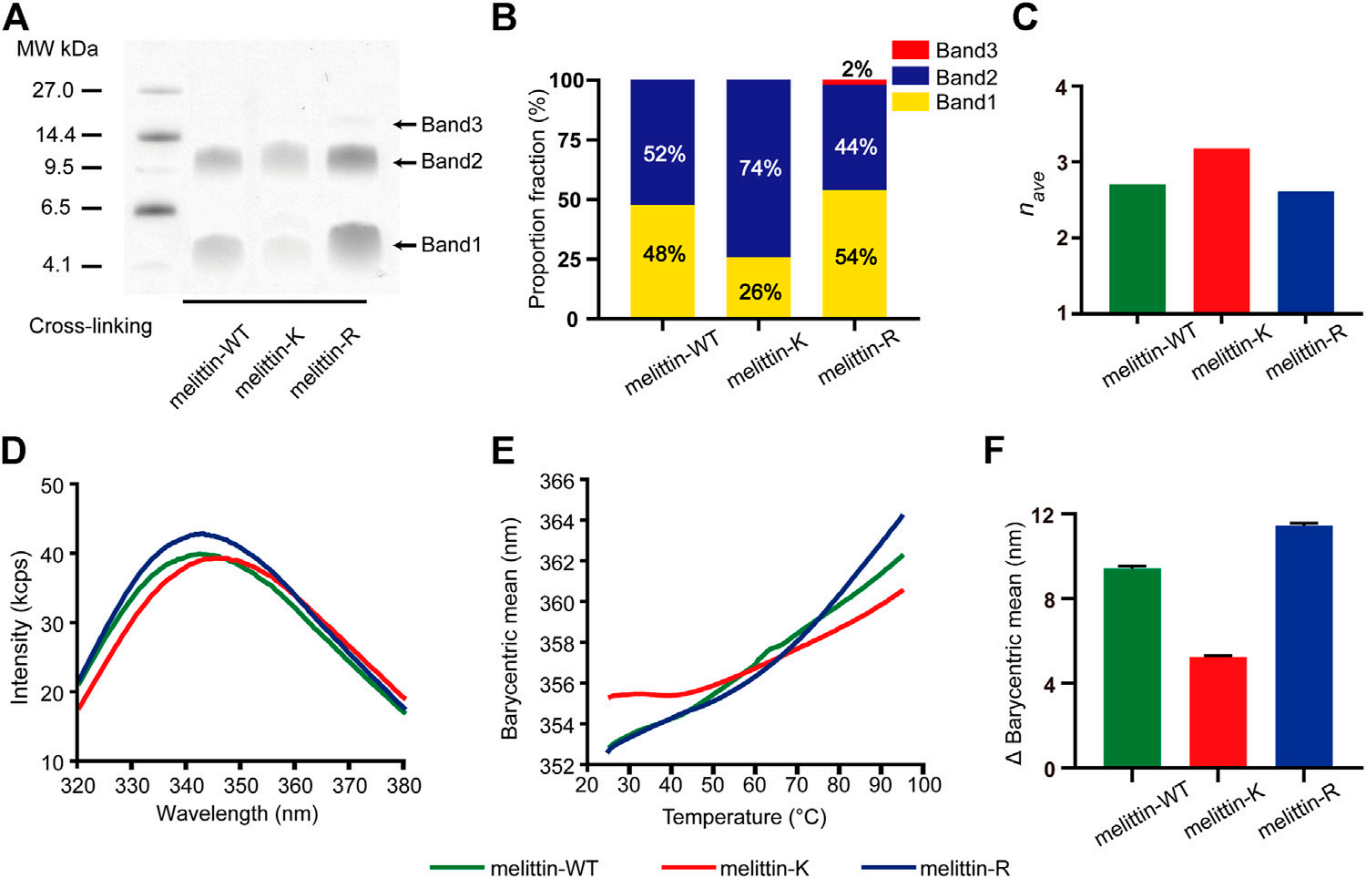
Impacts of K vs. R on the assembly structure of melittin. **(A)** SDS-PAGE of melittin peptides. Band 1, band 2, and band 3 are corresponding to dimer, tetramer, and hexamer, respectively. **(B)** The proportion fraction of band intensity (*p*_*i*_, i = 2, 4, and 6 for dimer, tetramer, and hexamer, respectively). **(C)** The averaged degree of oligomerization (*n*_*ave*_) of melittin peptides. **(D)** The tryptophan intrinsic fluorescence spectrum of melittin peptides at 25° C. **(E)** The barycentric mean of the melittin intrinsic fluorescence spectrum changes with temperature. **(F)** The variation of BCM from 25 to 95° C.

Previous X-ray crystallography and fluorescence experiments revealed that melittin-WT is driven by the hydrophobic interactions between the nonpolar patches to form a tetramer. To probe the impacts of immobilized cations on the assembly states of melittin in solution, we applied the PICUP method (Fancy and Kodadek, 1999; Bitan et al., 2001; Rahimi et al., 2009) to covalently stabilize the intermediate assemblies formed in a 200 mM melittin 1 × PBS solution, in which the solution condition is reported to favor the formation of melittin tetramer, and used SDS-PAGE to analyze the association states of melittins (Figure 2A;.Quay and Condie, 1983). The molecular weight of melittin peptide is 2846.5 g/mol (melittin-WT), 2790.5 g/mol (melittin-K), and 2930.5 g/mol (melittin-R). Three bands corresponding to dimers, tetramers, and hexamers were identified from the SDS-PAGE. This observation reveals that melittin-WT, -K, and -R form oligomers in bulk solution. Specifically, melittin-WT and melittin-K form dimers and tetramers, whereas melittin-R assembles into dimers, tetramers, and hexamers. The proportion of band intensity (*p*_*i*_, i = 2, 4, 6 for dimer, tetramer, and hexamer) obtained from SDS-PAGE images (Supplementary Figure S2; Supplementary Table S2) displays a dependence on the identity of immobilized cations (Figure 2B). For melittin-K, the tetramer was dominant (74% of the total population of melittin monomers), approximately three times as much as dimers. For melittin-WT and -R, dimer vs. tetramer was comparable in the population (WT: 48% vs. 52%; R: 54% vs. 44%). The proportion of band intensity (*p*_*i*_, where *i* = 2, 4, and 6 for dimer, tetramer, and hexamer, respectively) obtained from the SDS-PAGE image is positively proportional to the amount of peptide (de Moreno et al., 1986). It is worth to note that the Coomassie dyes can bind to every melittin peptide molecule in oligomers. The intensity of tetramer is four times as much as monomer and twice as much as dimer. Thus, the proportion of band intensity of dimer, tetramer, and hexamer should be calibrated by the degree of oligomerization (*n*_*i*_, where *i* = 2, 4, and 6 for dimer, tetramer, and hexamer, respectively) to obtain the proportion of oligomer. Thus, the averaged degree of oligomerization (*n*_*ave*_) is calculated as follows:

$$n_{ave}\;=\;\frac{1}{\sum \frac{p_{i}}{n_{i}}}$$

where *p*_*i*_ is the proportion of band intensity and *n*_*i*_ is the degree of oligomerization. The averaged degree of oligomerization of melittin peptides was determined to be 2.7 (melittin-WT), 3.2 (melittin-K), and 2.6 (melittin-R) (Figure 2C). Thus, the side chain of K facilitates melittin to form an oligomer with a higher assembly number rather than the side chain of R.

### Influence of K Vs. R on the Thermal Stability of Melittin

To understand the difference in the assembly structures of melittin-WT, -K, and -R, we compared the intrinsic fluorescence emitted by the tryptophan residue of melittin at 25 C. As shown in Figure 2D, the barycentric means (*BCM*, the wavelength corresponding to half of the peak area) of the peptide fluorescence spectroscopy was determined to be 352.6 nm for melittin-WT, 355.3 nm for melittin-K, and 352.6 nm for melittin-R. The variation in the *BCM* reflects the difference in the microenvironment of tryptophan. A higher *BCM* value is indicative of a more polar microenvironment surrounding the tryptophan. Furthermore, we evaluated the stability of melittin assemblies by measuring the change of intrinsic fluorescence upon an increased temperature from 25 to 95° C. As shown in Figure 2E, the *BCM* of melittin-WT shifted from 352.6 to 362.3 nm (the change in the *BCM*, Δ*BCM*, was 9.7 nm), and the *BCM* of melittin-R shifted from 352.6 to 364.1 nm (Δ*BCM* = 11.5 nm) upon thermal denaturation. In contrast, the *BCM* of melittin-K slightly increased from 355.3 to 360.5 nm (Δ*BCM* = 5.2 nm). The variations in the *BCM* values in the temperature range of 25–95°C are summarized and compared in Figure 2F. It leads to the finding that the tryptophan residues in the melittin-WT and -R oligomers are buried in the interior of oligomers at 25° C and become more solvent accessible upon heating. In contrast, the assembly structure of the melittin-K oligomer is relatively stable against thermal denaturation rather than melittin-WT and -R. Finally, we comment that no sign of large aggregate formation was observed, which is revealed by a parallel observation with the same set of melittin solutions by collecting the static light scattering (SLS) intensity at 266 nm (Supplementary Figure S3).

### Influence of K Vs. R on the Lipid Recognition of Melittins

The above results demonstrate that the immobilized cations exert opposite regulatory effects on the folding vs. assembly of melittin. The side chain of R is more favorable for the helical folding of melittin than the side chain of K, but the side chain of K is more favorable for the assembly of melittin than the side chain of R. The helical and assembly propensity of melittin may contribute to the penetration of melittin across the biological membranes (Yamashita et al., 2016; Hong et al., 2019; Oba et al., 2019). In the previous study, the guanidinium in the side chain of R is generally thought to be unique and crucial to induce the translocation of peptide across the cell membrane rather than the ammonium in the side chain of K, which is attributed to the hydrogen bonds and electrostatic interactions between guanidinium and phosphate groups, and the favorable interfacial energy between guanidinium and liposomal nonpolar domain (Tang et al., 2008; Wender et al., 2008; Yoo and Cui, 2010; Wexselblatt et al., 2014). Consequently, one may predict that melittin-R is more potent to interact and cross cell membranes than melittin-K. However, our observation that the effects of K vs. R on melittin folding are opposite to the effects of K vs. R on melittin assembly suggests that the outputs of melittin function responding to the cationic substitution between K and R could be complicated than the prediction that melittin-R possesses a stronger membrane penetrating ability than melittin-K. Thus, we tested the impacts of K vs. R substitution on modulating the interactions of melittin with lipid membranes by using two independent systems, an artificial liposome system and a naturally existing membranous system including bacteria and mammalian cells.

**FIGURE 3:**
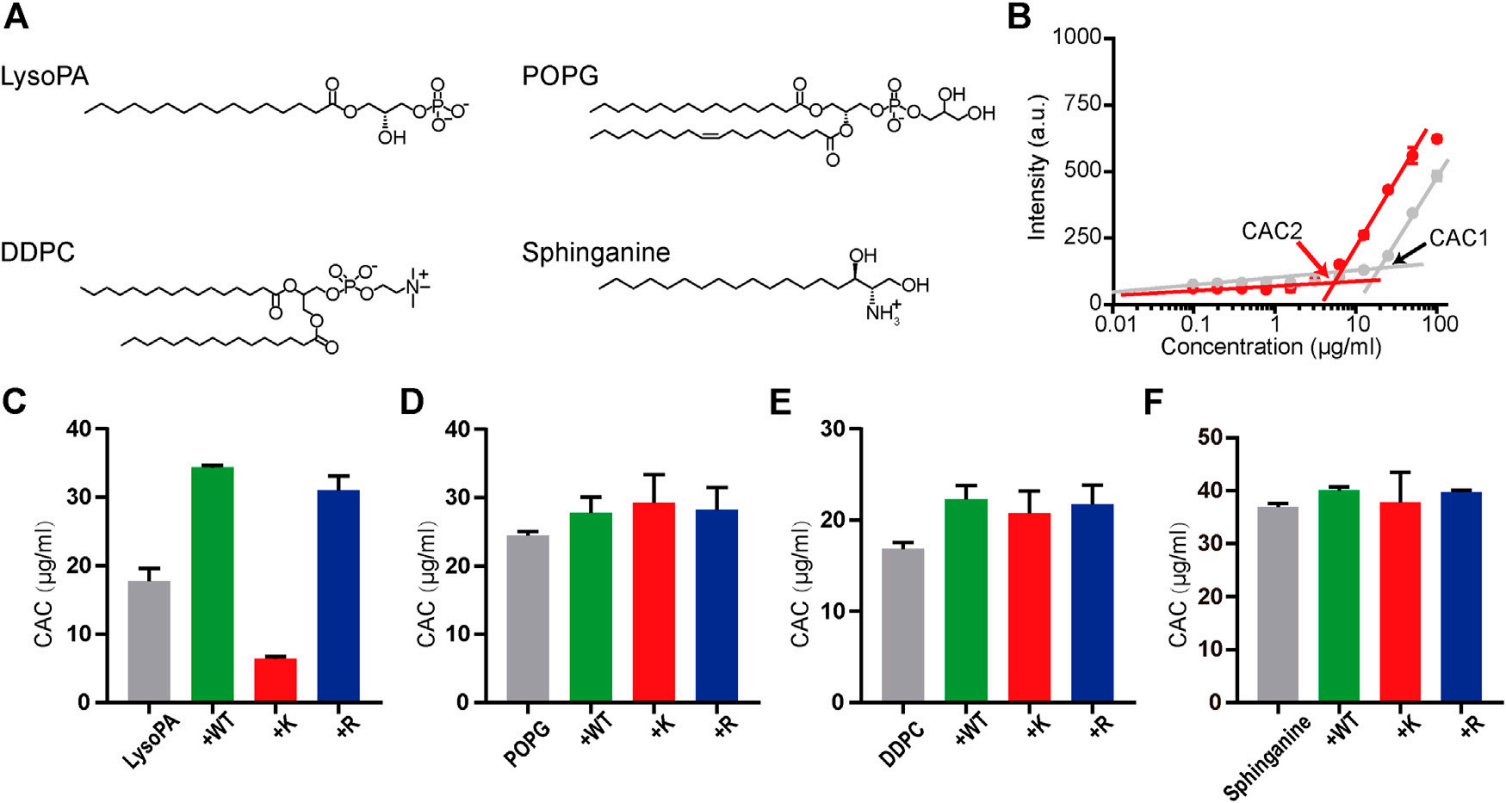
The molecular recognition of melittins with lipids. **(A)** The chemical structures of LysoPA, POPG, DDPC, and sphinganine. **(B)** The *CAC* values of LysoPA are determined from the DPH fluorescence data. The gray dots represent the DPH fluorescence of LysoPA at different concentrations, and the red dots represent the DPH fluorescence of lipids interacting with 6 μM melittin-K. **(C–F)** The *CAC* diagrams of different lipids interacting with melittin peptides. Data are presented as means ± SD (*n* = 3).

The interactions of melittin with liposome can be manifested by the change in the critical aggregation concentration (*CAC*) of lipid assembly responding to the addition of melittin. The *CAC* value is related to the assembly propensity of surfactant as follows:

$$\Delta G_{\mathrm{agg}}\;=\;-\mathrm{RT}\text{ln}\left(1\text{/}\mathrm{CAC}\right)\text{,}$$

where *R* is the universal gas constant, *T* is the absolute temperature, and Δ*G*_*agg*_ is the Gibbs free energy of aggregation (Jacob, 2011). We investigated the interactions of melittin with LysoPA (terminated with the phosphate group), POPG (terminated with the phosphoglycerol group), DDPC (terminated with the phosphocholine group), and sphinganine (terminated with the serinol group) (Figure 3A) by using a lipophilic dye, DPH, to determine the *CAC* of surfactants. The fluorescence intensity of DPH is low, when the concentration of the surfactant is below *CAC* and increases rapidly, when the concentration of the surfactant incrementally increases above *CAC* due to the uptake of DPH by the nonpolar membrane of surfactant micelles (Chattopadhyay and London, 1984). As shown in Figure 3B, the presence of 6 μM melittin-K decreased the *CAC* of LysoPA from 17.8 μg/ml (*CAC1*) to 6.4 μg/ml (*CAC2*), indicative of melittin-K interacting and strengthening the self-assembly propensity of LysoPA. As shown in the comparison of *CACs* of lipids with or without melittins (Figures 3C–F), the interactions between melittin and lipid exhibited a selectivity of the chemical identity of the lipid terminal group. When the terminal groups are phosphoglycerol, phosphocholine, or serinol, lipids are inert to interact with melittins, and the *CAC*s of POPG, DDPC, and sphinganine were almost unchanged upon the addition of melittins. In contrast, lipid terminated with phosphate is sensitive to melittin. The *CAC* of LysoPA, that is, 17.8 μg/ml, was increased to 34.4 μg/ml by adding 6 μM melittin-WT, and to 31.0 μg/ml by adding melittin-R. Different from the impact of melittin-R to disfavor the Gibbs free energy of PA assembly, the addition of 6 μM melittin-K decreased the CAC of LysoPA to be 6.4 μg/ml, favoring the Gibbs free energy of LysoPA assembly. These results demonstrate the molecular recognition of melittin with lipids can be modulated by the immobilized cations in peptides.

### Influence of K Vs. R on the Biological Activity of Melittins

**FIGURE 4:**
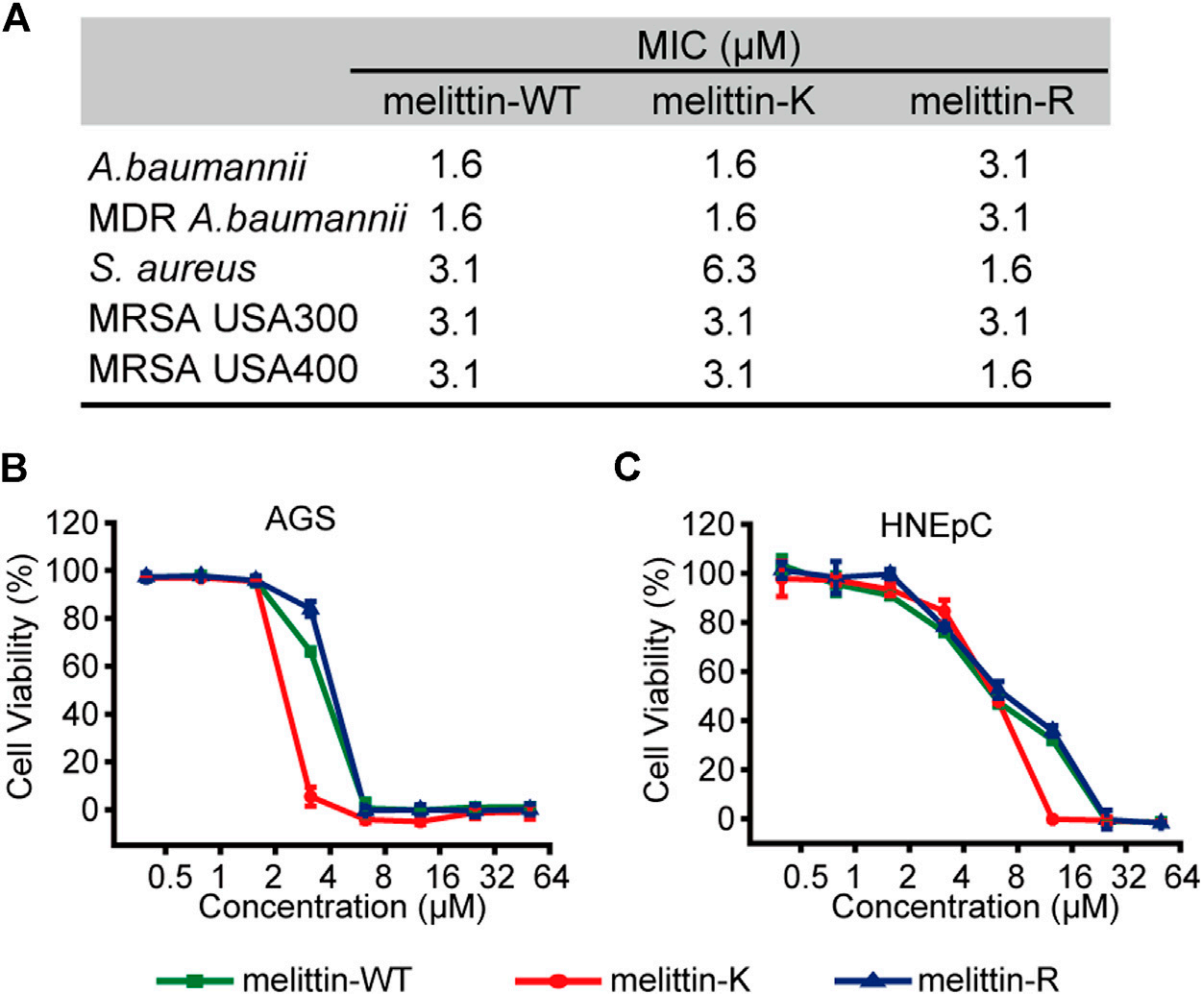
Biological activities of melittins. **(A)** The *MIC* values of melittins against Gram-negative bacteria (MDR *A. baumannii* 1814516 and *A. baumannii* BAA747) and Gram-positive bacteria (*S. aureus* 29231, MRSA USA-300, and MRSA USA-400). **(B–C)** The cytotoxicity of melittin peptides for AGS **(B)** and HNEpC cell lines **(C)**.

We evaluated the interactions of melittin with naturally occurred membranous systems by measuring the antibacterial activity and cytotoxicity of melittin. The antibacterial activity of melittins was assessed by a standard broth microdilution method in liquid culture. The bacterial strains used include Gram-negative bacteria (MDR *A. baumannii* 1814516 and *A. baumannii* BAA747) and Gram-positive bacteria (*S. aureus* 29231, MRSA USA-300, and MRSA USA-400). We compared the *MIC* values, the lowest peptide concentration at which 80% inhibition of bacterial growth, to depict the impacts of K vs. R on modulating the potential of melittin to interact and lyse bacterial membrane (Figure 4A). For Gram-negative bacteria, the *MIC*s of melittin-WT and -K (1.6 μM) were smaller than the MICs of melittin-R. For Gram-positive bacteria, melittin-WT and -K showed similar antibacterial activity against MRSA with the MICs of 3.1 μM. Interestingly, the MIC values of melittin-R for *S. aureus* 29231 and MRSA USA-400 were determined to be 1.6 μM, indicating its stronger potency to disrupt the bacterial membrane. We also evaluated the cytotoxcity to access the potency of melittin to interact and disrupt the eukaryotic cell membrane, including the cancer-originated cell AGS and normal tissue–originated cell HNEpC. Melittin-K exhibited higher potency in cytotoxicity (Figures 4B,C). Taken together, our results conveyed a message that the identity of cationic side chains is important and substitution between K and R side chains will result in a complicated change in the biological activity of melittin.

## Conclusion and Discussion

We have shown that the identity of immobilized kosmotropic vs. chaotropic cations, ammonium in the side chain of K vs. guanidinium in the side chain of R, is crucial to maintain the folding and assembly structure of melittin as well as the biological activity. A long-distance perturbation in the structural ordering of melittin has been identified by the comparison among the heterozygous wild-type melittin-WT and the homozygous kosmotropic melittin-K and chaotropic melittin-R. The influences of immobilized charges are divergent for the folding vs. assembly process. Specifically, the side chain of R promotes the helical formation of melittin rather than the side chain of K, following an inverse Hofmeister series: ammonium < guanidinium. In contrast, the side chain of K facilitates the assembly of melittin relative to that of R, showing a direct Hofmeister series: ammonium > guanidinium. The opposite trends of cationic substitution between K and R on the folding vs. assembly of melittin result in a fluctuation in the molecular recognition and biological functions of melittin.

Our observation that the side chains of K and R differ in their influence on the folding and assembly of melittin leads us to consider the differences in the pertinent properties of ammonium and guanidinium that contribute to the following intermolecular interactions. 1) Electrostatic interaction: The radius of guanidinium (4.4 Å) in water is larger than that of ammonium (1.4 Å), and thus the charge density of guanidinium is smaller than that of ammonium (Houriez et al., 2017). Ammonium is favorable to form electrostatic interactions relative to guanidinium. 2) van der Waals interaction: The polarizability of guanidinium is calculated to be greater than that of ammonium. The geometries of the two cations are also distinct because the guanidinium carbon is sp^2^-hybridized and the ammonium nitrogen is sp^3^-hybridized. The flat geometry provides guanidinium with π electrons which are easily displaced to achieve dispersion forces (Werner et al., 2014; Houriez et al., 2017). Consequently, guanidinium possesses a stronger potential to form van der Waals interactions and stacking interactions than does ammonium. 3) Hydrogen bond: Guanidinium is strongly hydrated than ammonium, and the enthalpy of hydration of guanidinium (602 kJ/mol) is more favorable than that of ammonium (−329 kJ/mol) (Werner et al., 2014). This trend in hydration strengthening because guanidinium forms more hydrogen bonds with water molecules than does ammonium (Werner et al., 2014). 4) Hydrophobic interaction: Both guanidinium and ammonium are discovered to play distinctive impacts on modulating the magnitude of hydrophobic interactions generated by the proximal nonpolar domain, which is spatially close to the cations. Ammonium favors hydrophobic interactions, while guanidinium diminishes hydrophobic interactions (Ma et al., 2015; Wang et al., 2017). Given that melittin is an amphiphilic peptide and driven by hydrophobic interactions to self-assemble, the divergent impacts of ammonium vs. guanidinium on hydrophobic interactions are needed to take into account for our experimental observation. The above analysis leads us to achieve two correlations. First, the immobilized guanidinium favoring the folding of melittin than ammonium is in line with the potential of cation to form van der Waals interactions and the hydrogen bonds with water. The hydrogen bonds with water are actually chaotropic for peptide folding, and thus the divergent impacts of immobilized ammonium and guanidinium on the folding of melittin are attributed to the different potential of immobilized cations to engage in van der Waals interactions. Second, the immobilized ammonium favoring the assembly of melittin than guanidinium is positively correlated with the ability of cation to form electrostatic interactions and their impacts on hydrophobic interactions. The previous X-ray crystallography with melittin revealed that hydrophobic interactions dominate the driving forces underlying melittin assembly, and thus we exclude the possible contribution of electrostatic interactions (Othon et al., 2009; Kurgan et al., 2019). Consequently, the immobilized ammonium vs. guanidinium impacts on melittin assembly that mainly reflects their divergent modulation effects on the hydrophobic interactions generated by the nonpolar domain of melittin.

Overall, the fundamental study with the immobilized cations on the folding and assembly of peptides highlights the complexity of the intermolecular interactions that govern biomolecular architecture, that is, both van der Waals and hydrophobic interactions exhibit context-dependent behaviors with the identity of immobilized charges. In the future, we will attempt to provide experimental evidence to elucidate the mechanism of how immobilized ions modulate van der Waals interactions and hydrophobic interactions. Such an effort will greatly enrich our knowledge about the design and manipulation of protein assembly and hydration behaviors.

## Data Availability

The original contributions presented in the study are included in the article/Supplementary Material; further inquiries can be directed to the corresponding author.
