# Supplementary material for: Opposite Regulatory Effects of Immobilized Cations on the Folding Vs. Assembly of Melittin
Source: Front Chem. 2021 Jun 11;9:685947. doi: 10.3389/fchem.2021.685947 (PMC8225954; doi:10.3389/fchem.2021.685947)
Supplement: Supplementary file 1 [file DataSheet1.docx]

Opposite Regulatory Effects of Immobilized Cations

on the Folding Versus Assembly of Melittin

Lanlan Yu^1^†, Zhun Deng^1^†, Wenbo Zhang^1^, Shuli Liu^2^, Feiyi Zhang^3^, Jianjian Zhou^4^, Chunhua Ma^4^, Chenxuan Wang^1^*

^1^ State Key Laboratory of Medical Molecular Biology, Institute of Basic Medical Sciences, Chinese Academy of Medical Sciences and Peking Union Medical College, Beijing 100005, China

^2^ Department of Clinical Laboratory, Peking University Civil Aviation School of Clinical Medicine, Beijing 100123, China

^3^ Institute for Advanced Materials, Jiangsu University, Zhenjiang 212013, China

^4^ Unchained Labs, Shanghai 201210, China

^†^Co-first authors

*** Correspondence:**Prof. Chenxuan Wang
email: [wangcx@ibms.pumc.edu.cn](mailto:wangcx@ibms.pumc.edu.cn)


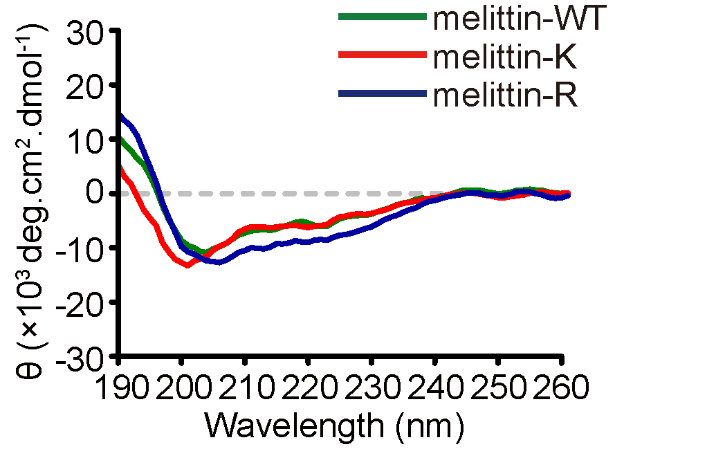


**FIGURE. S1** The CD spectra of melittin-WT, melittin-K, and melittin-R at 10 μM.


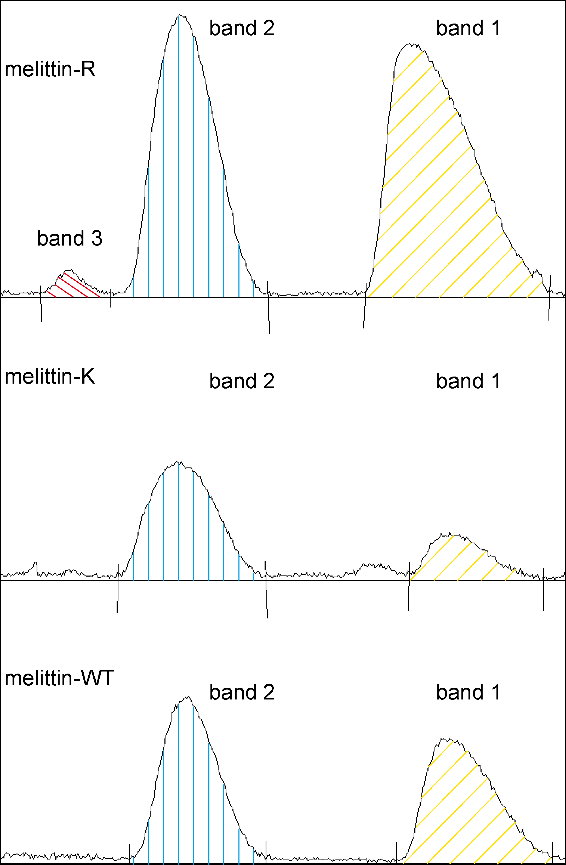


**FIGURE. S2** The analysis of band intensity in SDS-PAGE images.





**FIGURE. S3** The SLS intensity of melittin-WT, melittin-K, and melittin-R in a temperature range from 25 ℃ to 95 ℃.

**TABLE S1** The [*θ*]_222_/[*θ*]_208_ ratio of melittin-WT, -K, and -R.

| Concentration (μM) | [*θ*]_222_/[*θ*]_208_ ratio | | |
| --- | --- | --- | --- |
|  | melittin-WT | melittin-K | melittin-R |
| 200 | 0.83 | 0.84 | 0.88 |
| 50 | 0.76 | 0.63 | 0.81 |
| 25 | 0.74 | 0.56 | 1.29 |

**TABLE S2** The analysis of the proportion of band intensity obtained from SDS-PAGE images.

|  | band area | | | *p_i_* | | |
| --- | --- | --- | --- | --- | --- | --- |
|  | melittin-WT | melittin-K | melittin-R | melittin-WT | melittin-K | melittin-R |
| band 1 (dimer) | 13370 | 4316 | 32786 | 47.6% | 25.7% | 54.0% |
| band 2 (tetramer) | 14698 | 12481 | 26685 | 52.4% | 74.3% | 43.9% |
| band 3 (hexamer) |  |  | 1292 |  |  | 2.1% |
